# Fungus Ball in Concha Bullosa: A Rare Case with Anosmia

**DOI:** 10.1155/2013/920406

**Published:** 2013-07-08

**Authors:** Mahmut Özkırıs, Zeliha Kapusuz, Selda Seçkın, Levent Saydam

**Affiliations:** ^1^Department of Otolaryngology, Head and Neck Surgery, Bozok University Medical Faculty, Adnan Menderes Bulvarı No. 42, Yozgat, Turkey; ^2^Department of Pathology, Bozok University Medical Faculty, Adnan Menderes Bulvarı No. 42, Yozgat, Turkey

## Abstract

Concha bullosa is the pneumatization of the concha and is one of the most common variations of the sinonasal anatomy. The histopathological changes caused by the infections which arise from the impaired aeration of conchal cavity are frequently found. Fungus ball of the nasal cavity is an extremely rare, fungal infection with only three cases reported previously. In this paper, we present the fourth fungus ball case which developed within a concha bullosa and presented with anosmia.

## 1. Introduction

Fungus balls or mycetomas are extramucosal accumulations of degenerating fungal hyphae especially within chronically inflamed paranasal sinuses [1]. *Aspergillus* infections of the nose and paranasal sinuses are unusual but are being increasingly recognised in recent years. In the head and neck region, *Aspergillus* species can cause otomycosis, allergic paranasal sinusitis, invasive paranasal sinusitis, and aspergilloma of the paranasal sinuses [2]. In the English literature, we have found only three cases of fungus ball in concha bullosa published up to date [3]. In this case report, we describe a concha bullosa fungus ball in a 55-year-old woman. The clinical presentation, radiological and endoscopic findings, and management approach for this case are discussed. 

## 2. Case Report

**Figure 1:**
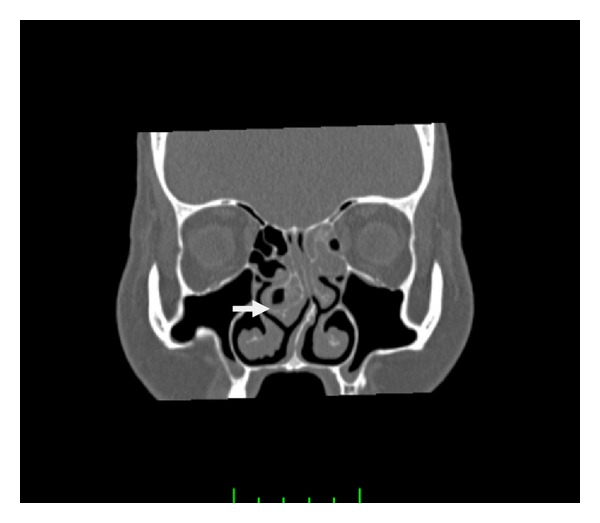
Coronal section of paranasal sinus on a noncontrast CT scan, showing partial heterogeneous opacification of the right concha bullosa, within which hyperdense foci are observed.

**Figure 2:**
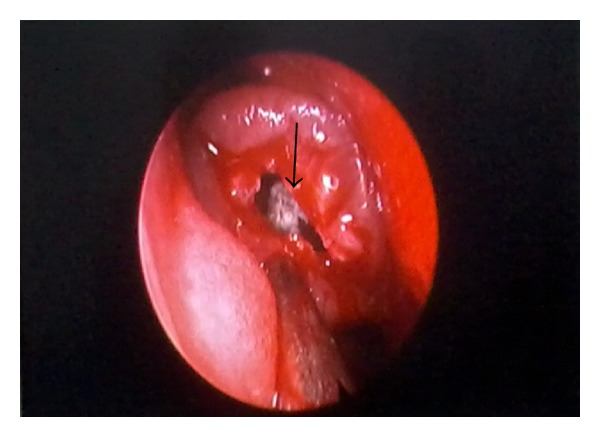
Endoscopic photograph showing a fungus ball in the right concha bullosa.

**Figure 3:**
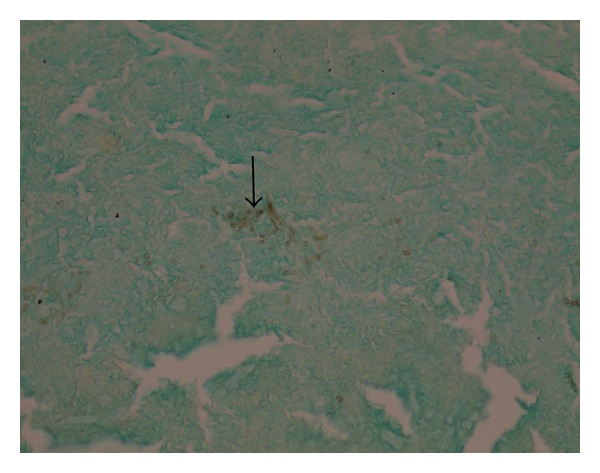
Histopathologic sections demonstrating fungi in Grocott method.

**Figure 4:**
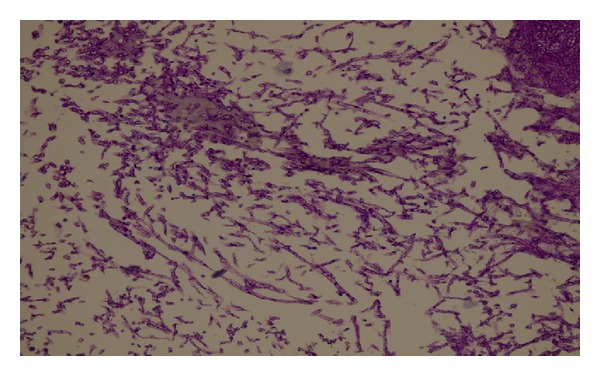
Characteristic septate hyphae of *Aspergillus* (Hematoxylin-EosinX400).

**Figure 5:**
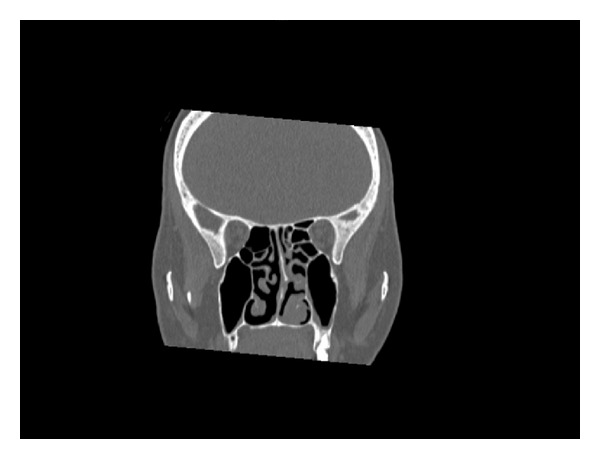
Coronal section of paranasal sinus CT scan of the patient after 1 month.

A 55-year-old woman was admitted to our clinic with 2-year duration of anosmia, nasal obstruction, and headache complaints. She had no history of nasal trauma, diabetes, immunosuppressive disease, and allergies. Nasal endoscopic examination revealed purulent nasal discharge in right nasal cavity, left septal deviation, and a hypertrophic right middle concha. The patient was otherwise healthy and results of routine laboratory tests were normal. Oral antibiotic (amoxicillin and clavulanic acid) and topical oxymetazoline hydrochloride 0.005% was prescribed for 10 days. Despite the treatment, patients' symptoms and endoscopic findings were not improved. A paranasal computed tomographic scan showed a right concha bullosa occluding the nasal passage near totally and left nasal septal deviation (Figure 1). There was no bone erosion and all the paranasal sinuses were well ventilated. She was scheduled for septoplasty and concha bullosa resection under general anesthesia. Following septoplasty the lateral lamella of right middle concha was excised. The conchal cavity was found to be filled with a mass which seems like a fungus ball in appearance (Figure 2). The mass and overlying mucosal layer was removed and sent for pathologic evaluation. The histopathologic examination with H&E staining revealed a proliferated fungal hyphal mass within the necrobiotic material. Fungal hyphae masses were stained positive with PAS and Grocott method for fungi. Hyphae masses consisted of thick septate filaments with narrow angle branching surrounded by inflammatory cells (Figures 3 and 4). The material sent for fungal culture was reported as negative. Endoscopic and paranasal CT examination 1 month after surgery showed completely open bilateral nasal passages with no sign of any infection (Figure 5).

## 3. Discussion

Concha bullosa occurs when the middle turbinate becomes pneumatized. This pneumatization results when ethmoid air cells migrate to the middle concha. As noted previously, this condition is a very common anatomic variation with a 14%–53.6% reported frequency by various studies [4]. The most common symptoms are nasal obstruction and facial pain. If the concha bullosa obstructs the middle meatus, the patient may develop sinusitis [5].

The recent rise in mycotic nasal and paranasal infections is due to both improved diagnostic ability with wide use of computerized tomography in sinonasal pathologies and an increase of the conditions that favor fungal infections such as immunosuppressive diseases and neoplastic conditions [6]. 

Fungal sinusitis has been classified in invasive and noninvasive forms, based on the presence or absence of hyphae in adjacent mucosa. Each group is subdivided into two subcategories, fulminant and chronic for the invasive form and fungus ball (previously called aspergilloma or mycetoma) and allergic for the noninvasive disease [7]. *Aspergillus* infections of the nose and paranasal sinuses are unusual but are being increasingly recognised in recent years [2]. In the head and neck region, *Aspergillus* species can cause otomycosis, allergic paranasal sinusitis, invasive paranasal sinusitis, and aspergilloma of the paranasal sinuses [2, 6]. Paranasal fungus balls are noninvasive lesions with no evidence of spread into or beyond the sinus mucosa. The most common causative agents are *Aspergillus *sp., mainly *A. fumigatus *and *A. flavus, *less frequently [8]. Cultures are often negative, and the fungus is identified in only 23% to 50% of cases, probably because of the low viability of the fungal components within the ball. Parallel to this data we could not be able to culture the fungal organisms in our case. Due to common appearance of *Aspergillus *sp., a fungus ball is often mischaracterized as aspergillosis or aspergilloma [7–9].

Conditions that favor fungal infections are diabetes, long-term treatments (antibiotics and cortisones), radio- and chemotherapy, immunosuppressive treatments, and immunodeficiency diseases [6]. Our patient had no history of diabetes, immunodeficiency, allergies, or any systemic disease. Many authors suggest that mycotic infections of the paranasal cavity are found more commonly in apparently healthy patients [8, 9].

From the imaging standpoint the radiological clues are apparent in all patients. Standard radiographs show unilateral partial or complete opacification of a sinus, most often the maxillary sinus. Areas of hyperdense well-defined foci are observed in 25% to 50% of cases, strongly suggesting the diagnosis of a sinus fungus ball [9, 10]. Macroscopically, the fungus ball appears friable and grumous, may be green, yellow brown, or black, and detaches easily from the mucosa [11]. Characteristic CT scan findings include opacification with a hyperdensity in its core, osteosclerosis, and osteolysis [7–9]. Magnetic resonance imaging is often used for further evaluation of affected areas when aggressive surgical intervention is planned. A differential diagnosis has to be kept in mind, including bacterial sinusitis, mucocele, malignant tumour, or metastasis [8]. In our patient, a paranasal computed tomographic scan showed a right concha bullosa occluding the nasal passage near totally and left nasal septal deviation. There was no bone erosion. Definitive diagnosis is based chiefly on the macroscopic appearance and histopathology, as cultures are frequently negative (70% of the cases) [12]. Invasive forms are also determined by histopathologic examination. Microscopic appearance revealed hyphal intrasinusal mass without invasion resembling aspergillus morphology stained by PAS and Grocott method also [13].

Treatment consists of endoscopic nasal surgery, and the technique used is dictated by the location of the fungus ball (concha bullosa resection, middle antrostomy, ethmoidectomy, and sphenoidotomy). Treatment in asymptomatic patients is generally recommended as well; however, there is little evidence to support this approach. The complication rates of these procedures are low, with exceptionally high cure rates, and postoperative or perioperative antifungal treatment is not warranted for noninvasive fungal sinusitis [14].

The present case, to the best of our knowledge, is the fourth patient with fungus ball in concha bullosa. In conclusion, clinicians should be aware that a chronic rhinosinusitis that is unresponsive to normal management with a radiodense focus is highly suggestive of fungus ball.
